# Early-stage mapping of macromolecular content in APP^NL-F^ mouse model of Alzheimer’s disease using nuclear Overhauser effect MRI

**DOI:** 10.3389/fnagi.2023.1266859

**Published:** 2023-10-09

**Authors:** Anshuman Swain, Narayan D. Soni, Neil Wilson, Halvor Juul, Blake Benyard, Mohammad Haris, Dushyant Kumar, Ravi Prakash Reddy Nanga, John Detre, Virginia M. Lee, Ravinder Reddy

**Affiliations:** ^1^School of Engineering and Applied Science, University of Pennsylvania, Philadelphia, PA, United States; ^2^Center for Advanced Metabolic Imaging in Precision Medicine, Perelman School of Medicine, University of Pennsylvania, Philadelphia, PA, United States; ^3^Department of Neurology, Perelman School of Medicine, University of Pennsylvania, Philadelphia, PA, United States; ^4^Center for Functional Neuroimaging, Perelman School of Medicine, University of Pennsylvania, Philadelphia, PA, United States; ^5^Center for Neurodegenerative Disease Research, Perelman School of Medicine, University of Pennsylvania, Philadelphia, PA, United States; ^6^Alzheimer’s Disease Research Center, Perelman School of Medicine, University of Pennsylvania, Philadelphia, PA, United States

**Keywords:** NOE, Alzheimer’s disease, multipool fitting, lipid dyshomeostasis, CEST

## Abstract

Non-invasive methods of detecting early-stage Alzheimer’s disease (AD) can provide valuable insight into disease pathology, improving the diagnosis and treatment of AD. Nuclear Overhauser enhancement (NOE) MRI is a technique that provides image contrast sensitive to lipid and protein content in the brain. These macromolecules have been shown to be altered in Alzheimer’s pathology, with early disruptions in cell membrane integrity and signaling pathways leading to the buildup of amyloid-beta plaques and neurofibrillary tangles. We used template-based analyzes of NOE MRI data and the characteristic Z-spectrum, with parameters optimized for increase specificity to NOE, to detect changes in lipids and proteins in an AD mouse model that recapitulates features of human AD. We find changes in NOE contrast in the hippocampus, hypothalamus, entorhinal cortex, and fimbria, with these changes likely attributed to disruptions in the phospholipid bilayer of cell membranes in both gray and white matter regions. This study suggests that NOE MRI may be a useful tool for monitoring early-stage changes in lipid-mediated metabolism in AD and other disorders with high spatial resolution.

## Introduction

Alzheimer’s disease (AD) is the leading cause of dementia in adults aged 65 and older, with an estimated 6.7 million people in America currently living with the disorder across all ages (Alzheimer’s disease, 2023). People with AD exhibit increased memory impairment as well as changes in non-memory domains, including executive function and language processing (Knopman et al., 2021). Despite the prevalence of AD and concerted efforts to understand its etiology and symptoms, there remains a lack of robust techniques to detect early-stage (i.e., preclinical stage) AD and its progression (Chandra et al., 2019). Magnetic resonance imaging (MRI) is the most commonly used clinical tool to evaluate patients with brain disorders as the technique is noninvasive and provides excellent soft tissue contrast with high spatial resolution (Chandra et al., 2019). Clinical scans typically employ T_1w_ and T_2w_ contrasts that can distinguish between gray matter, white matter, and CSF. In particular, T_1w_-MRI can be used to detect regional brain atrophy associated with neurodegeneration. Additional information about white matter tracts can be obtained using diffusion-weighted imaging (DWI) methods. Coupled with machine learning and deep learning, these methods have shown remarkable results in classifying healthy, AD, and mild-cognitive impairment (MCI) patients.

Although strategies of diagnosing AD with MRI seem promising, structural MRI scans do not provide information about molecular changes in AD, while deep learning techniques are less sensitive to classifying early-stage Alzheimer’s and require substantial domain knowledge for interpretability and translation (Tanveer et al., 2020; Zhang et al., 2022). Positron emission tomography (PET) is another imaging tool that can provide information on metabolism, with decreased regional glucose metabolism as observed by FDG-PET providing clinical indications of AD pathology. In addition, molecular PET agents for amyloid and tau further serve to identify if patients with cognitive complaints are likely to have AD and to track and predict neurodegeneration, respectively. Despite its clinical utility and specificity, PET is limited largely by its spatial resolution (~4–5 mm) in addition to non-specific tissue uptake of certain radiotracers, complex quantitative analyzes, and the cumbersome synthesis and immense costs of multi-tracer studies (Marcus et al., 2014; Bao et al., 2021).

Simple, yet informative, techniques for assessing metabolic, related to glucose and neurotransmitter metabolism, and macromolecular content, related to lipid and protein content, of brain tissue include chemical exchange saturation transfer (CEST) and nuclear Overhauser effect (NOE) MRI (Van Zijl and Yadav, 2011; Jones et al., 2013; Benyard et al., 2023). A typical CEST experiment involves the saturation of exchangeable metabolite protons at a resonance frequency offset from that of water protons. The frequency offset is determined by the chemical shifts of these exchangeable protons, which typically correspond to amine, amide, and hydroxyl groups of biomolecules. During the saturation period, labile protons exchange with water protons, thus attenuating the bulk water signal which can subsequently be measured using conventional MRI sequences. Saturation transfer MRI has been used to determine regional changes in metabolite content of preclinical models of AD and human subjects. The primary CEST contrasts used to detect macromolecular and metabolic changes in AD include amide-proton transfer (APT)-CEST, GluCEST, glucoCEST, and CrCEST, which are sensitive to protein, glutamate, glucose, and creatine content, respectively. Studies employing these contrasts have found regional changes, primarily in the hippocampus, in AD pathology (Orzylowska and Oakden, 2021).

NOE is another saturation transfer method involving saturation of non-exchangeable species which are typically the methyl and methylene protons of lipids and proteins. The saturated protons transfer energy to nearby exchangeable protons, termed a “relayed NOE (rNOE)” effect, which then subsequently exchange with water and attenuate the bulk water signal. NOE has been used to measure changes in lipid and protein content with one recent study assessing changes in NOE contrast in a mouse model of AD. That study used ultra-short echo time (UTE) imaging following a saturation module to reduce effects of direct saturation and magnetization transfer on the observed NOE contrast while increasing sensitivity to short T_2_ species corresponding to proteins (Orzylowska and Oakden, 2021). However, NOE contrast is observed primarily through exchange effects with bulk water, which does not necessitate the need for a UTE readout and the NOE-UTE magnetization asymmetry ratio (MTR_asym_) contrast observed in that study more closely resembles that of typical MT measures, which show sensitivity to myelin macromolecular content in the brain.

The current study uses a standard spoiled gradient-echo (GRE) readout with saturation parameters sensitive to NOE contrast to acquire images from offsets ranging from-100 to +100 ppm, generating a well-characterized Z-spectrum that provides information on multiple metabolites and macromolecules. MTR_asym_ analysis and Lorentzian fitting, along with an atlas-based analysis, are used to characterize changes in these metabolites in the APP^NL-F^ knock-in mouse model of Alzheimer’s disease.

## Methods

### Animal preparation

The study was performed using 6- to 8-month-old C57BL/6 J (wild-type (WT), *n* = 5) mice and APP^NL-F^ knock-in (AD, *n* = 5) mice using a protocol approved by the IAUCAC committee of the University of Pennsylvania. Prior to the MRI experiments, the mouse was anesthetized using 1.5% isoflurane and its head was placed in a conical head restrainer. A respiratory pillow pad and rectal probe were used to monitor the breathing rate and body temperature, respectively, of the mouse throughout the MRI experiments. The conical restrainer was placed in a 20 mm diameter ^1^H transceiver volume head coil (m2m Imaging) and MRI experiments were performed on a 9.4 T horizontal magnet interfaced with an Avance III HD console (Bruker BioSpin, Germany).

### MRI experiments

#### Anatomical image acquisition

A localizer was acquired first, followed by a T_1_-weighted FLASH (TE/TR = 4/498 ms, four averages, 16 slices) and T_2_-weighted RARE (TE_1_/TE_2_ = 33/121 ms, TR = 3,082 ms, two averages, 16 slices, rare factor = 6). A 1 mm thick slice located 3.3 mm anterior to lambda was selected as our slice of interest, and localized shimming and post-processing registration/segmentation was performed on this slice.

#### Steady-state NOE MRI acquisition

For NOE MRI acquisitions, a water saturation shift referencing (WASSR) image (TE/TR = 4/410 ms, 22 frequency offsets from 0 to 1 ppm with a step-size (ss) of 0.1, B_1_ = 0.1μT, one average) was acquired for correcting B_0_ inhomogeneities (Kim et al., 2009). A full Z-spectrum was acquired for the saturated images with 174 offsets variably spaced as follows: 0-6 ppm (ss = 0.1), 6-10 ppm (ss = 0.5), 10-20 ppm (ss = 1 ppm), and 20-100 ppm (ss = 10 ppm). The acquisition parameters included: TE/TR = 4/3010 ms, B_1_ = 1.0 μT, saturation duration (T_sat_) = 3.0 s, and two averages. An unsaturated image (with the same parameters as the saturated images, except offsets of ±300 ppm) was additionally acquired. The FOV was 20 mm x 20 mm with an image matrix size of 128 × 128, resulting in an in-plane resolution of 0.156 mm x 0.156 mm for all images.

#### NOE_MTR_ analysis

NOE_MTR_ images were calculated using the following equation:


$$NOE_{MTR}\left(\%\right)=\frac{\left(S_{-300ppm}-S_{-3.5ppm}\right)}{S_{-300ppm}}\;x\;100\%$$

where S_-3.5ppm_ and S_-300ppm_ represent the magnitude signal of an image voxel at offsets of-3.5 ppm and-300 ppm, respectively. NOE_MTR_ was calculated after correcting for B_0_ inhomogeneities and compared to NOE_MTR_ calculated *without* correcting for B_0_ inhomogeneities.

#### Lorentzian fitting of Z-spectrum

In addition to NOE_MTR_, the Z-spectrum of each voxel was fit using multi-pool Lorentzian fitting with five pools of interest corresponding to direct saturation (DS), magnetization transfer (MT), amines, amides, and relayed NOE. Multi-pool fitting is accomplished by characterizing the Z-spectrum as a sum of Lorentzian functions, as defined by the following equation:


$$L\;\left(\omega_{n},a_{n},\sigma_{n}\right)=\overset{N}{\underset{n=1}{\sum }}a_{n}\frac{\sigma_{n}}{\sigma_{n}-4{\left(\omega -\omega_{n}\right)}^{2}}=1-\frac{S}{S_{0}}$$

where ɷ_n_, α_n_, and σ_n_ represent the frequency offset, amplitude, and full width at half-maximum of each pool. The initial parameter estimates used for each pool were based on prior estimates in Zaiss et al. (2011). Before fitting, Z-spectra were denoised by organizing the Z-spectrum from each voxel into a Casorati matrix and performing singular value decomposition (SVD). The threshold for choosing the *n* singular values was determined by the median criterion and the denoised Casorati matrix was finally reshaped to recover the images at each offset (Breitling et al., 2019). To decrease the computational time of fitting each pixel, the fitting was parallelized. Maps of each contrast were generated using the amplitude of the Lorentzian fit to each pool.

#### Post-processing/ROI selection

**Figure 1:**
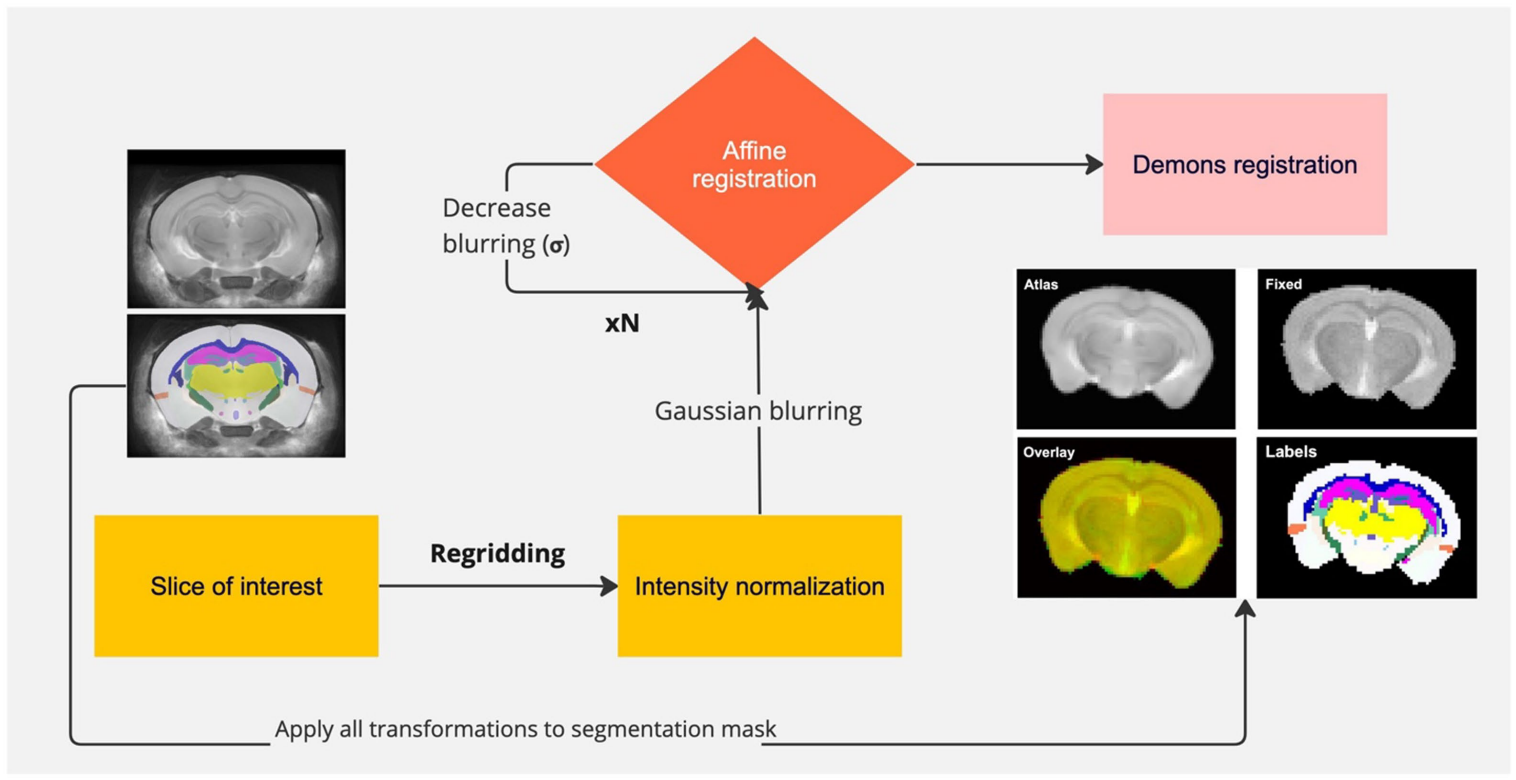
Pipeline of registration and segmentation of a mouse brain. The slice of interest is selected from the atlas, re-gridded to match the size of the fixed image, and intensity normalized to the intensity of the fixed image. Gaussian blurring is used to find a coarse affine transformation of the atlas to the fixed image, followed by subsequent iterations in which blurring is reduced to achieve finer transformations. The last transformation involves non-linear registration of the atlas to the fixed image. All transformations are applied to the segmentation with nearest-neighbor interpolation. Representative registered atlas and labels are shown.

All image processing steps were performed in MATLAB 2022a (MathWorks, CA). For post-processing, automated skull-stripping was performed on the T_2_w images using a fuzzy c-means clustering with three different clusters. For automated registration, a C57BL/6 J mouse brain atlas from Dorr et al. (2008) was used as a template. The template consisted of a three-dimensional T_2_w RARE image and corresponding brain segmentations for the entire mouse brain. Since our acquisitions were single-slice experiments, the corresponding brain slice was determined by comparing the sagittal slice of the localizer to the middle sagittal slice of the atlas. Using the brain lambda as the fiducial marker, the atlas slice was determined to be-3.4 mm away from the center of lambda. The T_2_w image from each acquisition was intensity matched to the atlas image using a histogram-based normalization. The atlas and fixed images were then heavily blurred by a Gaussian filter and subsequently registered using an affine transformation. This process was repeated for a sequence of 10 iterations, with the σ of the Gaussian kernel reduced to allow for registration of finer features. The final transformation was a non-linear registration using the Demons algorithm (Vercauteren et al., 2009). The corresponding segmentations from the atlas were registered by applying the transforms determined from the anatomical image registration, albeit using nearest-neighbor interpolation of pixels to prevent creation of non-integer labels following the transformations. Registration and segmentation accuracy were evaluated by the user. Figure 1 shows a schematic of the pipeline used to register and segment the mouse brain, along with a representative example of the registered atlas and labels for the corresponding mouse brain slice. The gray and white matter regions of interest that were evaluated were as follows: gray–cerebral cortex, entorhinal cortex, hippocampus, thalamus, hypothalamus and white–corpus callosum, cerebral peduncle, fimbria. The mean pixel values of each region of interest (ROI) for NOE_MTR_ (B_0_-corrected and-uncorrected) and the four pools of interest–rNOE, amine, APT, and MT–from Z-spectrum fitting were calculated.

### Statistical analysis

A linear mixed effects model was used in which the fixed effect was disease type (whether the mouse was a wild-type or AD model) and the random effect was the ROI to determine if there was a significant difference between wild-type and AD mice. To determine if disease type had a different effect for each ROI, the intra-class correlation coefficient was calculated. Ordinary least squares (OLS) regression models were fit to each ROI to determine which ROIs were significantly affected by disease type. The alpha value was set to 0.05 for all measures.

## Results

**Figure 2:**
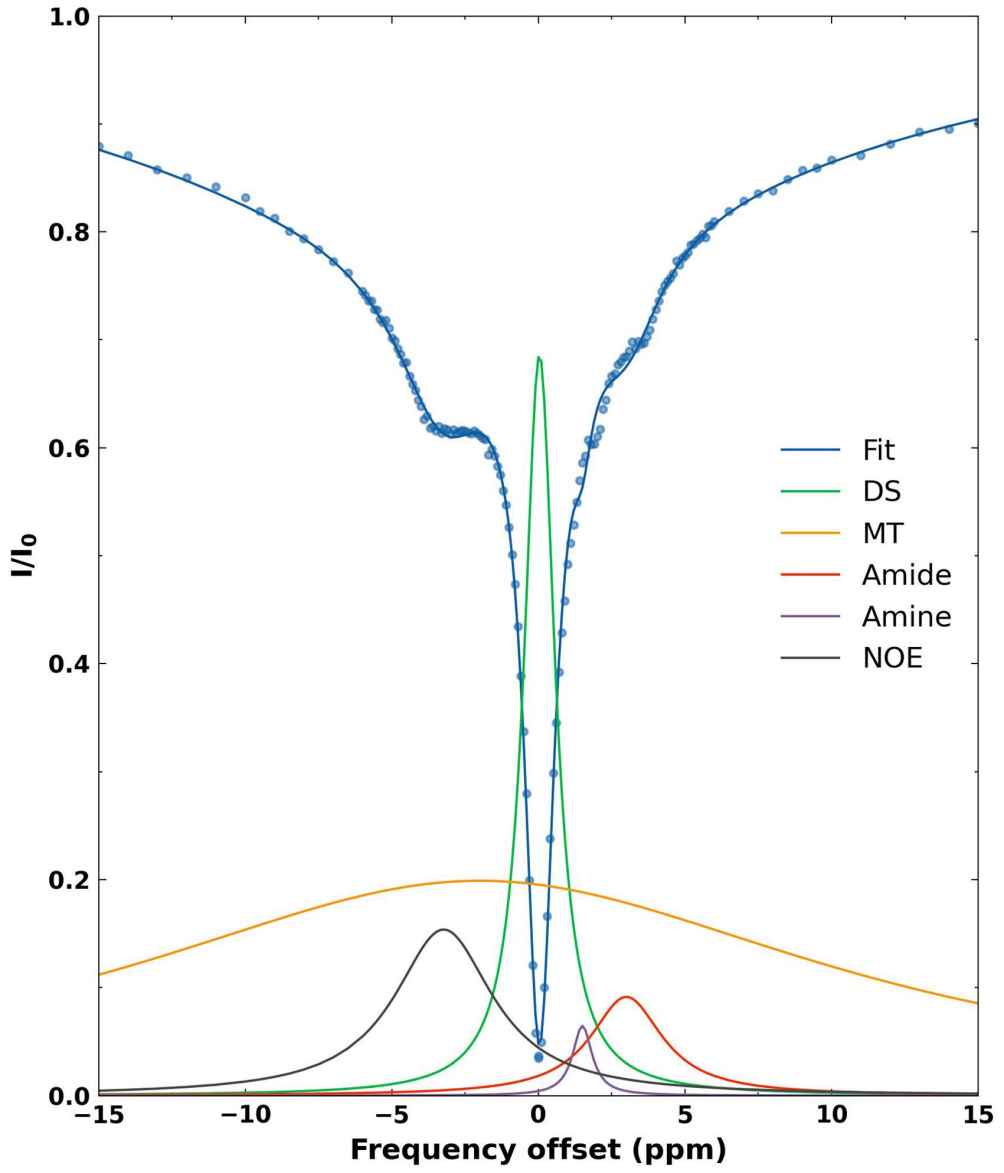
Representative fitted Z-spectrum (blue curve) from the whole brain of a WT mouse. The blue scatter points represent mean normalized intensity values at each offset. The five fitted metabolite pools are as follows: DS (green), MT (yellow), amide (red), amine (purple), NOE (black).

A representative Z-spectrum and its corresponding fit are shown for the average pixels from the whole brain of a WT mouse (Figure 2). Five Lorentzian line shapes are seen corresponding to the five pools of interest. At-3.5 ppm, corresponding to the selected frequency offset for NOE there is a 58% contribution from combined DS + MT (52% + 6%) effects and a 40% contribution from NOE effects. The remaining 2% results from the tails of the peaks corresponding to amine and amide.

**Figure 3:**
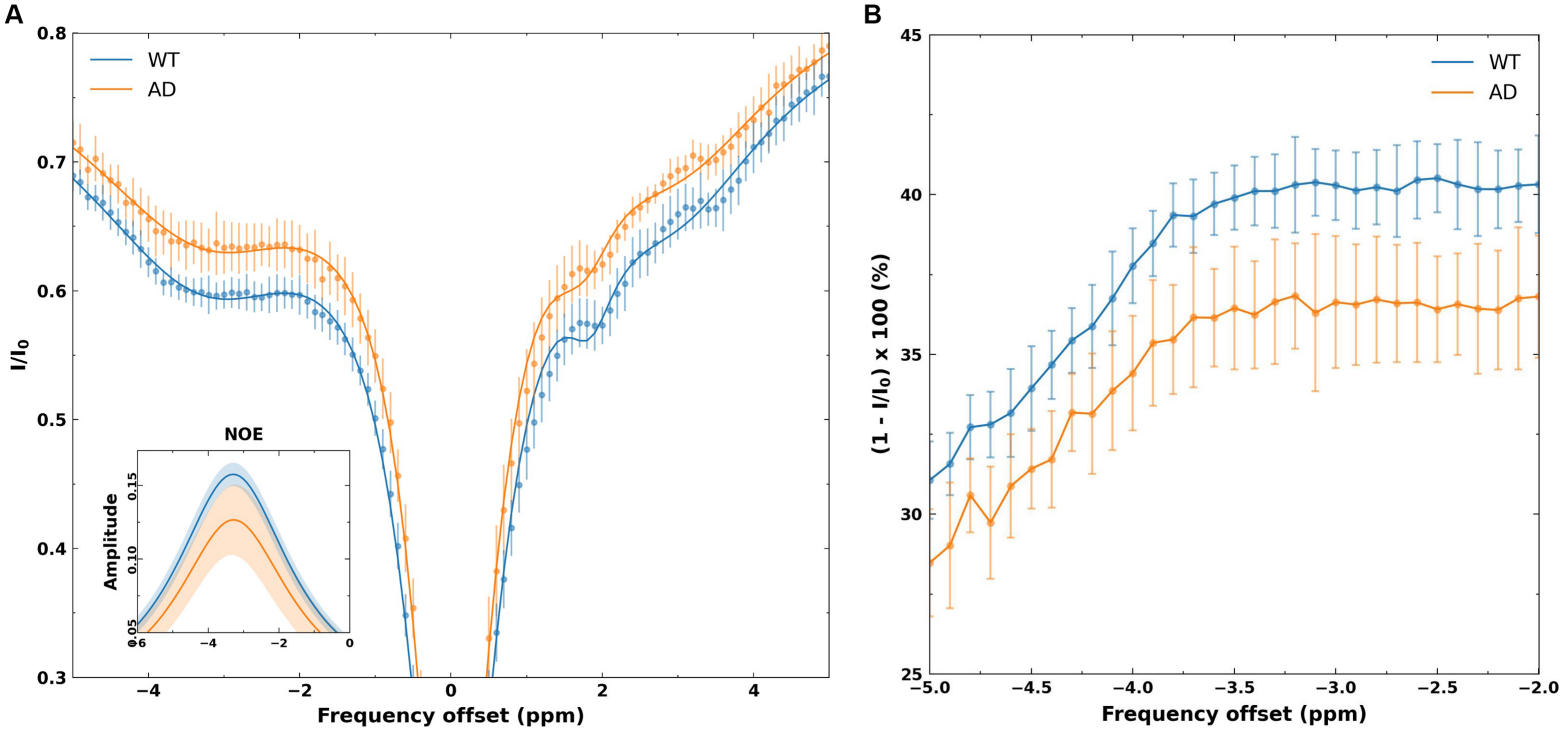
**(A)** Z-spectra from the hippocampus of WT and AD mice. The points represent mean normalized intensity values for each group for offsets of −5 to 5 ppm, with error bars representing the standard deviation for each offset. The inset shows the fitted NOE amplitudes derived from the multi-pool fitting, with the WT group showing a higher amplitude than the AD group. The shaded regions in the inset represents the standard deviations of the fits in each group. **(B)** The MTR_asym_ spectra from the hippocampus of WT and AD mice. As shown, −2 to −3.5 ppm shows a flat profile, likely reflecting the broad line shape of macromolecules that undergo cross-relaxation. The shift of-3.5 ppm is chosen for NOE metrics as this corresponds to macromolecular shifts observed in high-resolution ^1^H NMR at 3.5 ppm upfield of water.

**Figure 4:**
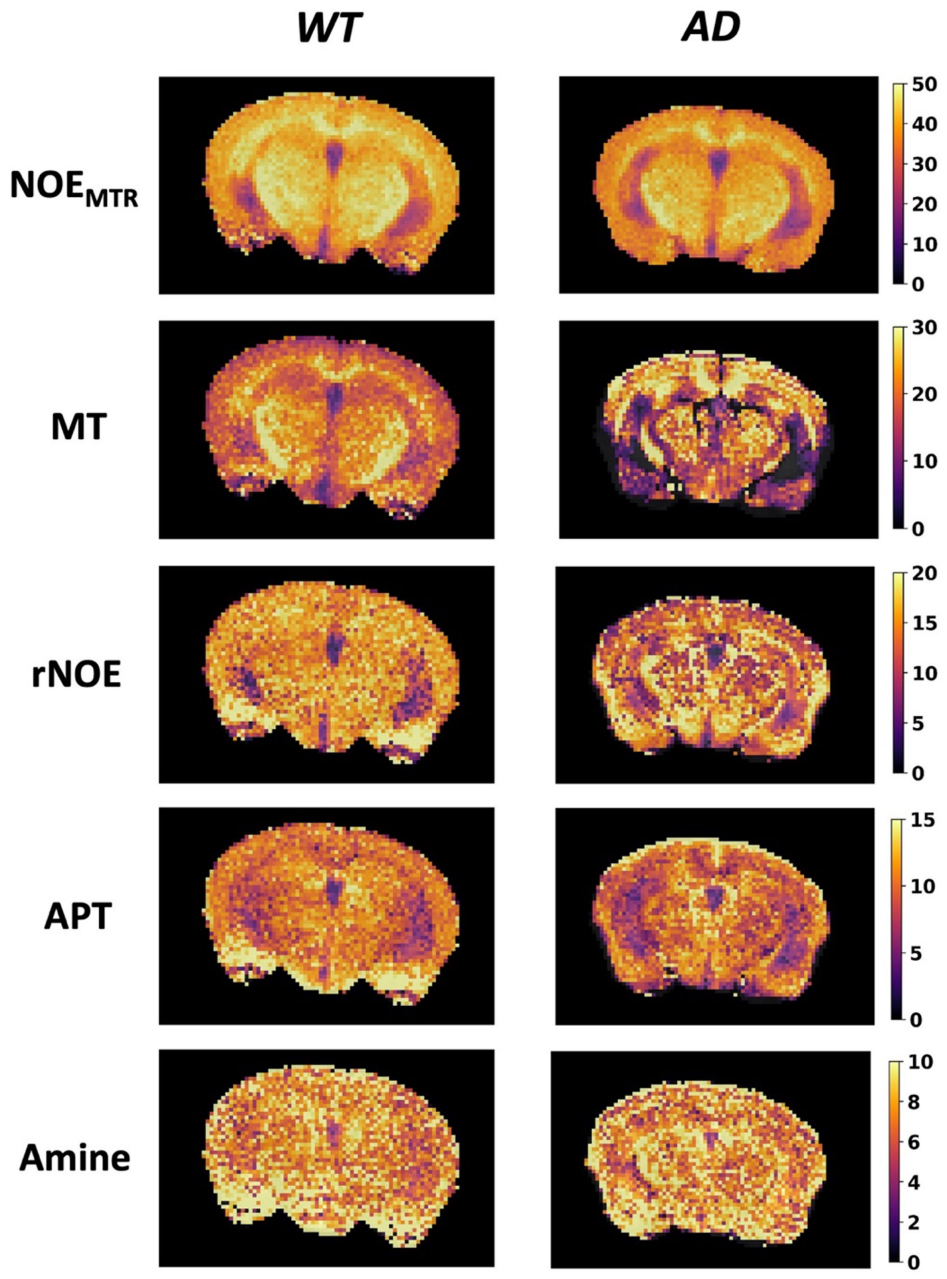
Representative global maps from a WT and AD mouse. The first row shows the NOE_MTR_ contrast generated using Equation (1), while the subsequent rows are generated from pixelwise multi-pool fitting of Z-spectra as described by Equation (2). The colormaps are in units of %.

Figure 3A shows the mean Z-spectrum from the hippocampus of a WT and AD mouse. As shown in the inset, there is a substantial decrease in rNOE contrast, derived from multi-pool fitting, between WT and AD mice in the hippocampus. The MTR_asym_ curve for NOE_MTR_ from the hippocampus of a WT and AD mouse (Figure 3B) shows that both rNOE and NOE_MTR_ decrease in the hippocampal region for AD mice. Figure 4 shows representative global maps from WT and AD mice for all pools of interest in addition to NOE_MTR_, with a visually apparent reduction in all contrasts following AD pathology, particularly in the cerebral cortex, hippocampus, and thalamic regions.

**Figure 5:**
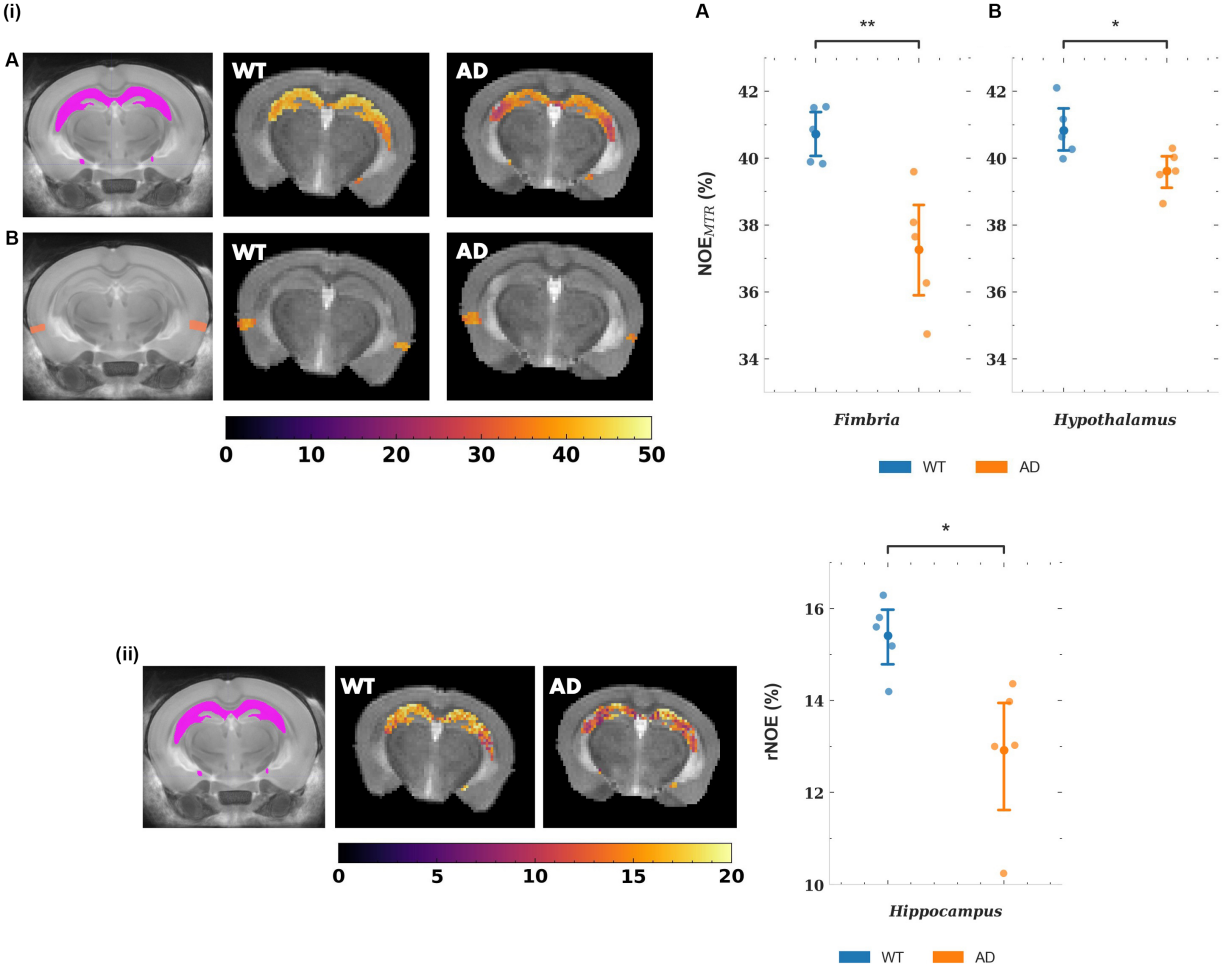
(i) Representative NOE_MTR_ maps from the segmented hippocampus **(A)** and entorhinal cortex **(B)** of a WT and AD mouse. There is a statistically significant decrease in NOE_MTR_ observed in the hippocampus and entorhinal cortex of the AD mouse. The point plots show the average NOE_MTR_ from each region for WT and AD mice. (ii) Representative relayed NOE maps from the hippocampus of a WT and AD mouse. Similar to NOE_MTR_, there is an observable decrease in relayed NOE contrast in the hippocampus of the AD mouse which shows statistical significance as seen in the point plot to the right. The colormaps are in units of %. ** = *p* < 0.01, * = *p* < 0.05.

**Figure 6:**
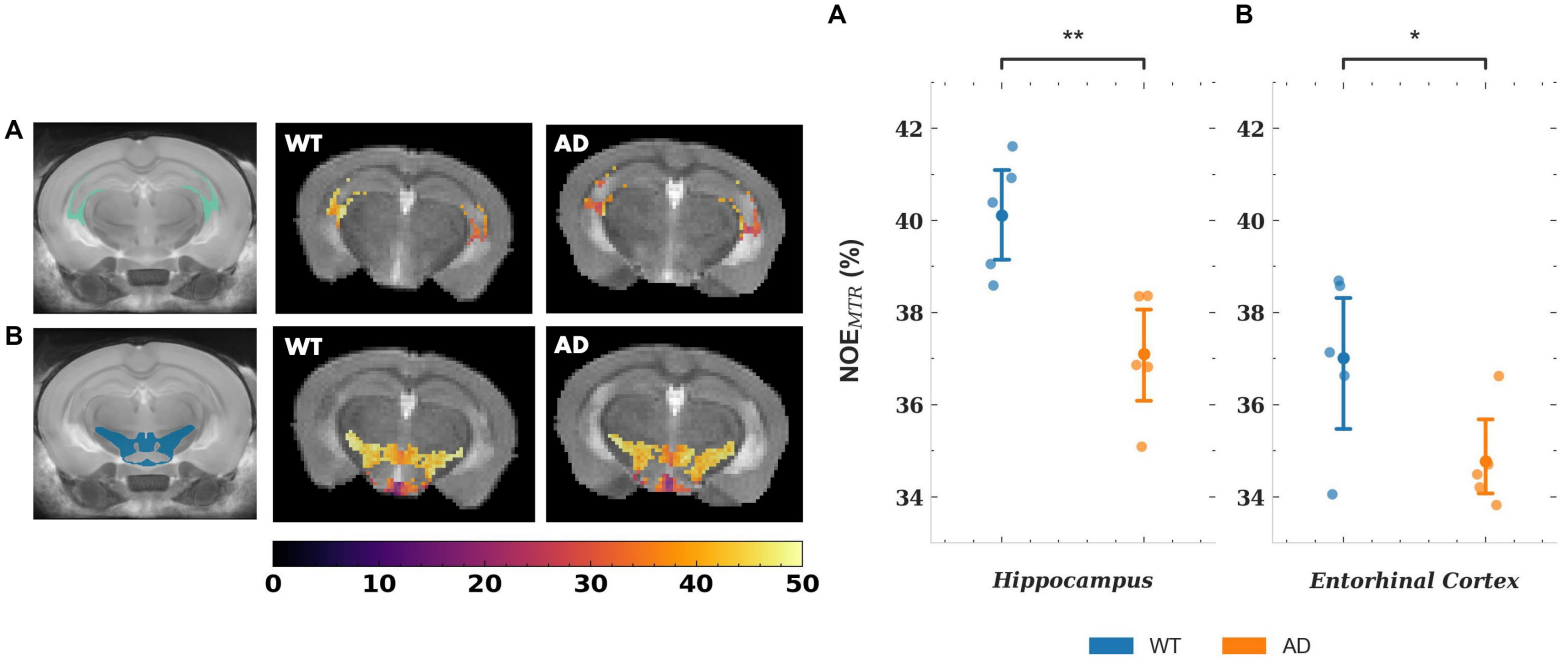
Representative NOE_MTR_ maps from the fimbria **(A)** and hypothalamus **(B)**. A statistically significant drop is observed in AD mice compared to WT mice, as shown by the point plots to the right. The point plots represent the average NOE_MTR_ in each region for WT and AD mice, with an *n* = 5 in both groups. In addition, the representative maps show observable changes in contrast, with the fimbria showing a strikingly lower contrast in the AD mouse. The colormaps are in units of %. ** = *p* < 0.01, * = *p* < 0.05.

The mean NOE_MTR_ values for a subset of gray matter regions that showed statistically significant changes are plotted in Figure 5, along with representative segmented maps from WT and AD mice for the corresponding regions. For the hippocampus and entorhinal cortex, the decreases in mean NOE_MTR_ of the regions between WT and AD are as follows: hippocampus – 40.1 ± 1.3 v. 37.1 ± 1.4% and entorhinal cortex – 37.0 ± 1.9 v. 34.8 ± 1.1%. Based on the linear mixed effects model, the reduction in the hippocampus (~3%) showed strong statistical significance (*p* < 0.01) while the reduction in the entorhinal cortex (~2.2%) showed statistical significance, but to a lesser degree (*p* < 0.05). The mean rNOE value, determined by Lorentzian fitting, also showed a significant decrease (*p* < 0.05) in the hippocampus (15.4 ± 0.8 v. 12.9 ± 1.6%) as is visually apparent in the representative NOE maps of WT and AD mice. Although the hippocampus was the only region with a statistically significant change, the mean rNOE values for all regions decreased in AD mice. Figure 6 shows mean NOE_MTR_ values and corresponding segmented regions for the hypothalamus and fimbria. The hypothalamus shows a decrease from 40.8 ± 0.8 to 39.6 ± 0.6% (*p* < 0.05), and the fimbria shows a strong statistically significant decrease from 40.7 ± 0.8 to 37.3 ± 1.8% (*p* < 0.01). Furthermore, although the scope of this work focuses on NOE metrics, the amine and amide pools (figure not shown) presented statistically significant changes in the hippocampus between WT and AD mice, with a decrease of 8.0 ± 0.5% v. 7.4 ± 0.2% and 10.2 ± 1.0% v. 8.8 ± 0.3%, respectively (*p* < 0.05 for both pools).

**Figure 7:**
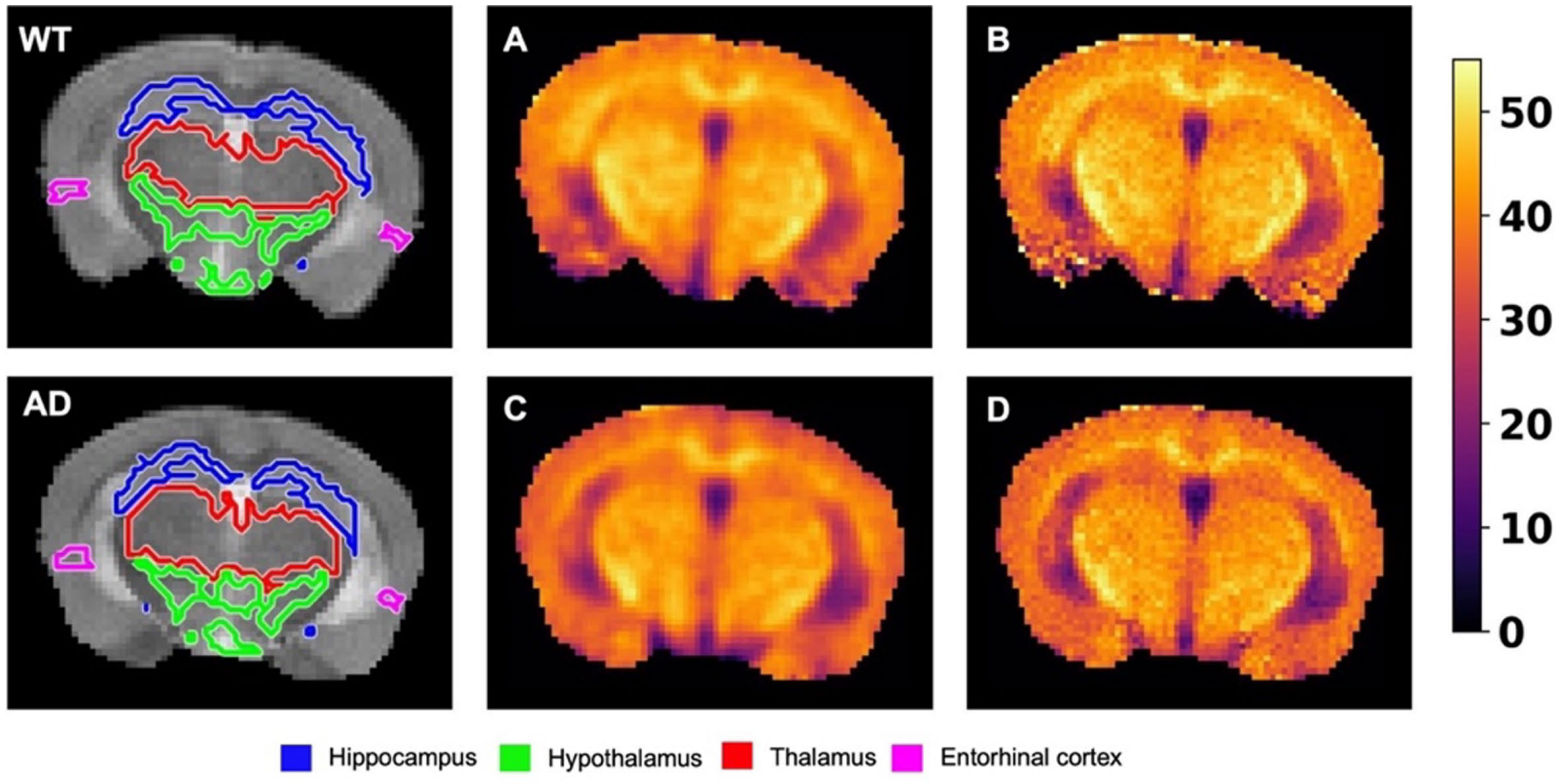
The leftmost image of the top and bottom rows shows representative T_2w_ images from a WT and AD mouse brain, respectively. The images are overlaid with their respective segmentations, with the colors of the regions corresponding to the labels listed on the bottom. **(A)** The B_0_ uncorrected NOE_MTR_ map from the corresponding WT mouse brain following a Gaussian blurring with *σ* = 0.75. **(B)** The B_0_ corrected NOE_MTR_ map with no filtering applied. **(C)** and **(D)** correspond to **(A)** and **(B)** respectively, but for the corresponding AD mouse brain. The regions affected by B_0_ correction are the thalamus and entorhinal cortex, while the hippocampus and hypothalamus remained unaffected. The thalamus showed statistically significant differences between WT v. AD but lost its statistical significance post-B_0_ correction, while the entorhinal cortex showed the opposite effect. The colormaps are in units of %.

Figure 7 shows the same representative NOE_MTR_ maps from a WT and AD mouse as in Figure 4, along with representative NOE_MTR_ maps that have not undergone B_0_ correction but have undergone denoising with a Gaussian filter. Visually, the B_0_ corrected and denoised B_0_ uncorrected images look similar, with changes in contrast most apparent in the thalamus as B_0_ correction seems to produce more homogeneous pixels in this region. Following quantitative ROI analysis for the uncorrected images, the thalamus shows a drop in NOE_MTR_ contrast in AD with strong statistical significance (*p* < 0.01). The hippocampus and hypothalamus also show statistically significant drops in AD, similar to the B_0_ corrected NOE_MTR_ maps (*p* < 0.01 and *p* < 0.05, respectively). However, only the B_0_ corrected NOE_MTR_ maps show a significant change in the entorhinal cortex.

## Discussion

In this study, we investigated changes in NOE_MTR_ and rNOE between wild-type and APP^NL-F^ knock-in mouse models of Alzheimer’s disease. We evaluated these metrics using MTR_asym_ analysis and multi-pool Lorentzian fitting for different regions of interest. We found a statistically significant decrease in NOE_MTR_ in the hippocampus, hypothalamus, entorhinal cortex, and fimbria of AD mice. In addition, we found a statistically significant decrease in rNOE in the hippocampus of AD mice. The observed signal changes are likely attributed to changes in macromolecular content during early-stage AD pathology.

Since exchange with bulk water mediates the signal in NOE MRI, the formation of Aβ oligomers and plaques may be a likely cause of the signal reduction that we observe in the AD mouse models. However, cross-relaxation from the fatty acid chains of lipid moieties must also be considered. More recently Zhao et al. (2023), proposed that the majority of relayed NOE signal observed at-3.5 ppm arises from the methylene (-CH_2_) and methyl (-CH_3_) protons of lipid side chains in phospholipid membranes. Consequently, the changes in NOE_MTR_ and rNOE observed in this study may arise primarily from lipid changes, as opposed to protein contributions from Aβ deposition. Furthermore, the decrease in signal is higher for rNOE than APT which suggests that rNOE and APT signals are not necessarily correlated and arise from different macromolecular structures (i.e., lipids v. proteins, respectively).

The changes in lipid metabolism are complex in AD, with aberrant lipid metabolism proposed to not only initiate, but result from, the buildup of Aβ plaques (Kang and Rivest, 2012; Kao et al., 2020). Lipid profiling techniques, such as matrix-assisted laser desorption/ionization-time of flight (MALDI-TOF) and liquid chromatography-mass spectrometry (LC–MS), are typically used to characterize lipids with tremendous specificity (Kofeler et al., 2012). Although these techniques provide exhaustive characterization of lipids, changes in lipid composition across different regions of the brain and their effects on cognitive function vary greatly in AD. In the hippocampal region, clinical studies have shown decreased levels of docosahexaenoic acid (DHA), a fatty acid that provides structural integrity to neuronal cells. In addition, experimental reduction of DHA in primate and rodent models has shown deterioration in cognitive and behavioral scores (Cunnane et al., 2013). Fourier transformed infrared microspectroscopy (FTIR) shows increased oxidized lipid concentration surrounding the cores of Aβ plaques (Benseny-Cases et al., 2014; Sanchez-Molina et al., 2020). A well-observed phenomenon following the cleavage of APP to Aβ monomers and oligomers is the disruption of neuronal cell membranes. Mrdenovic et al. have shown that Aβ plaques and soluble Aβ oligomers interact with the hydrophobic cell membrane core, causing changes in the orientation of the phospholipid side chains and reducing their mobility (Mrdenovic et al., 2020). In addition, Aβ monomers cause dehydration of the phospholipid heads, reducing regions of exchange with surrounding water. Aβ has also been shown to affect cholesterol integrity and content in cell membranes, further affecting membrane structure and mobility.

Given changes in the cell membrane lipid content and integrity in AD, the changes observed by NOE MRI are more likely driven by changes in lipid content. Since cross-relaxation effects are driven by the mobility of spins, altered conformations and reduced mobility of phospholipid side chains due to Aβ buildup and cholesterol changes would reduce NOE. In addition, oxidation of lipids can reduce the number of -CH_2_ protons that participate in cross-relaxation, further reducing the NOE effect. Interestingly, the amplitude of the amide pool derived from the multi-pool fitting, corresponding to tissue protein content, shows a lower amplitude than rNOE. This suggests that lipids may contribute more signal to rNOE than proteins.

Although lipid dysfunction may be the primary cause of the observed changes in rNOE signal, changes in protein content may also provide confounding effects. Protein content is known to be affected in Alzheimer’s disease due to the buildup of Aβ fibrils. These fibrils form due to the cleavage of amyloid precursor protein (APP) by β - and γ -secretases to form Aβ-peptides. These peptide monomers self-associate into various assemblies, with dense, insoluble amyloid fibrils and soluble oligomers being the primary types (Chen et al., 2017). Although the various types of Aβ assemblies are difficult to characterize, the common structural elements include an extended or beta sheet structure with main chain hydrogen bonding that is resistant to exchange (Kheterpal et al., 2003). These exchange-resistant protons are likely the cause of the reduction in the APT signal observed in the AD mouse model, since APT relies on the exchange of amide proton groups, which comprise the peptide bonds of proteins, with bulk water. In addition, the reduction in amine signal, likely from guanidyl and amine functional groups on proteins, may be caused by reduced exchange due to the packed and relatively immobile conformation of Aβ peptides, reducing exposure of labile amine protons to bulk water.

The changes observed in NOE_MTR_ in different brain regions of AD mice agree well with prior findings using structural MRI as well as CEST. CEST studies have shown decreased APT contrast in AD mouse models, attributed to inaccessible exchangeable protons in the beta sheet conformation of Aβ fibrils (Wells et al., 2015; Huang et al., 2022). In addition, structural MRI has shown significant gray matter atrophy of the hippocampus in AD mice (Ni, 2021). Prasad et al. (1998) have reported a decrease in membrane phospholipids in the hippocampus of AD patients, which corroborates the reduced rNOE in the hippocampus of AD mice. In the another study Zheng et al. (2018), report a decrease in choline and choline-related metabolites in the hypothalamus of 5 month-old APP/PS1 mice, which may indicate impairments in cell membrane phospholipid synthesis and cell apoptosis (Michel et al., 2006). The reduction in choline is associated with amyloid pathology which, as described earlier, disrupts lipid metabolism and cell membrane mobility, thus reducing NOE effects. The limitations of previous preclinical studies using CEST and NOE MRI included the lack of atlas-based analyzes of mouse brain. As a result, the observed NOE_MTR_ change in the entorhinal cortex of an AD mouse observed in this study presents a novel finding, as per our knowledge. In fact, recent studies show that alterations in the entorhinal cortex, whose function is to relay information between the hippocampal formation and neocortex, are present in early stages of AD (Igarashi, 2023).

In addition to changes in gray matter regions, NOE_MTR_ shows a significant change in the fimbria. The fimbria is a white matter bundle that connects the hippocampus to the fornix and thus to the rest of the brain. Alterations of the fimbria in AD are not well-characterized, while changes in the hippocampus and fornix are well-studied with hippocampal atrophy and neuronal loss in the fornix being known effects of AD pathology (Mielke et al., 2012). Recently, quantitative susceptibility mapping (QSM) has shown changes in the fimbria of subjects with AD. The increased susceptibility is attributed to a decrease in the diamagnetic protons of lipids and proteins comprising the myelin sheaths or an increase in paramagnetic species due to iron deposition (Au et al., 2021). Given these competing effects determined by QSM, and NOE MRI’s sensitivity to lipids, the reduction in NOE_MTR_ signal may be attributed primarily to the breakdown of myelin lipids. Studies using myelin water imaging, a well-established method for quantitatively assessing myelin integrity, show changes in myelin in the cerebral white matter following aging (Faizy et al., 2020) and decreases in myelin content in late myelinating brain regions such as the frontal white matter and corpus callosum in AD (Dean et al., 2017; Lim et al., 2022). As reported in juvenile rats (Downes and Mullins, 2014), the fornix is one of the last brain regions to achieve complete myelination, and given hypotheses regarding late-myelinating regions as the first areas to be implicated in AD, the decrease in NOE_MTR_ in the fimbria may be reflective of pathological changes in preclinical AD. In addition, myelin water fraction (MWF), as derived from myelin water imaging, decreases in individuals with mild-cognitive impairment (MCI), suggesting that demyelination plays a role in preclinical stages of AD (Bouhrara et al., 2018). Demyelination also leads to more rapid cognitive decline in cognitively unimpaired individuals (Gong et al., 2023).

NOE MRI shows strong sensitivity to alterations in gray matter and white matter integrity in 6 to 8-month-old early-stage AD mice. In addition, the APP^NL-F^ knock-in mouse model is a non-aggressive model of AD, reflective of the pre-clinical stages of AD in human subjects (Sasaguri et al., 2017). However, NOE_MTR_ is limited in its specificity as the metric is comprised of DS, MT, and relayed NOE effects. Although contributions of direct saturation are low at-3.5 ppm, it is difficult to determine changes in MT separate from changes in NOE. Multi-pool Lorentzian fitting increases the dynamic range of NOE MRI by allowing the extraction of multiple pools, thus allowing for the separation of MT and NOE. In this study, only the hippocampus showed significant changes in rNOE following the multi-pool fit, likely associated with changes in lipid content and potential contributions from Aβ deposition. Although multi-pool fitting increases the dynamic range, it requires extensive scan times to acquire a full Z-spectrum and can thus be difficult to implement clinically. Given that MT is sensitive to macromolecular content and has shown specificity to white matter regions in the brain, the combination of rNOE and MT in NOE_MTR_ can provide a joint metric that reflects changes in the gray and white matter regions of the brain. B_0_ correction serves to denoise the image as the correction process follows a polynomial or spline interpretation over a range of offsets, inadvertently denoising the image. In this study, the thalamus and entorhinal cortex were two regions that were affected by B_0_ correction, in which the statistical significance changed for both regions following B_0_ correction. The loss of statistical significance in the thalamus is explained by the relatively inhomogeneous pixel intensities, which become more homogeneous and of lower intensity following correction. For the entorhinal cortex, the gain in statistical significance appears following B_0_ correction since the cerebral cortex suffers the most from B_0_ field inhomogeneities due to brain-skull interfaces, and thus susceptibility effects. Since discrepancies exist following B_0_ correction, this post-processing step may be necessary to acquire more reproducible and reliable data. However, acquisition of an entire Z-spectrum is unnecessary if there are sufficient offsets acquired around the offset of interest to perform a well-posed interpolation. Acquiring fewer offsets and correcting for field inhomogeneities allows for robust NOE_MTR_ analysis in a clinically feasible scan time.

A limitation of this study is the long scan time needed to acquire a full Z-spectrum, which poses a challenge in translating to clinical use. However, rapid-acquisition techniques using compressed sensing, parallel imaging, and magnetic resonance fingerprinting (MRF) may allow for acquiring fully sampled Z-spectra in clinically feasible scan times (Heo et al., 2017; Cohen et al., 2018). Another limitation of this study is the use of a sub-optimal B_1_ saturation power for APT- and amine-weighted contrasts. Prior studies have found that a B_1_ of 2.0 μT provides the best and most interpretable contrast for comparing healthy to pathological tissue, primarily for tumor and stroke (Dou et al., 2019; Zhou et al., 2019). However, a thorough analysis has not been performed for the optimal B_1_ power in relation to AD pathology. Given that NOE is the main focus of this work, optimizing B_1_ power for APT and amine contrast is reserved for future studies. Furthermore, another limitation of this study is the separation of NOE_MTR_ contributions from MT versus rNOE. rNOE is shown to be more sensitive to membrane lipids but lacks sensitivity to white matter myelin lipids as their fatty acid backbones have very limited mobility, decreasing the NOE effect. Consequently, NOE_MTR_ changes in the gray matter likely arise from rNOE changes, while changes in that of white matter may arise primarily from MT changes with smaller, additional changes in rNOE. Further studies are needed to validate the sources of rNOE from different regions of the brain, perhaps by using suitable model systems that exhibit Aβ buildup and lipid dyshomeostasis separately. This would greatly improve the specificity of NOE MRI and allow for increase clinical translation in assessing microstructural changes in AD and other neurodegenerative disorders.

## Conclusion

This study used NOE MRI to assess early-stage changes in the APP^NL-F^ knock-in mouse model of Alzheimer’s disease. Using NOE_MTR_, significant changes in gray matter regions central to early-stage AD pathology were observed, specifically the entorhinal cortex. Consequently, the NOE_MTR_ metric in the entorhinal cortex can serve as a potential biomarker for detection of AD. In addition, NOE_MTR_ and rNOE metrics showed significant changes in the hippocampus, which is a region well-known to be affected by AD. As NOE is sensitive to macromolecular content in the brain, these metrics can serve to assess disrupted cell membrane lipid integrity in early stages of AD, serving as another potential biomarker for detection of AD. Given changes observed in the fimbria by NOE_MTR_, this metric may show sensitivity for white matter bundles and can potentially be used for assessing white matter changes in AD as well. Overall, NOE MRI showed good sensitivity to pathological regions in AD and can plausibly be used to map early-stage changes in AD pathology.

## Data availability statement

The raw data supporting the conclusions of this article will be made available by the authors, upon reasonable request.

## Ethics statement

The animal study was approved by Institutional Animal Care and Use Committee of the University of Pennsylvania.

## Author contributions

AS: Writing – original draft. NS: Writing – review & editing. NW: Writing – review & editing. HJ: Writing – review & editing. BB: Writing – review & editing. MH: Writing – review & editing. DK: Writing – review & editing. RN: Writing – review & editing. JD: Writing – review & editing. VL: Writing – review & editing. RR: Conceptualization, Writing – review & editing.

## References

[ref1] Alzheimer’s disease (2023). 2023 Alzheimer's disease facts and figures. Alzheimers Dement. 19, 1598–1695. doi: 10.1002/alz.13016, PMID: 36918389

[ref2] Au C. K. F. Abrigo J. Liu C. Liu W. Lee J. Au L. W. C.. (2021). Quantitative susceptibility mapping of the hippocampal Fimbria in Alzheimer's disease. J. Magn. Reson. Imaging 53, 1823–1832. doi: 10.1002/jmri.27464, PMID: 33295658

[ref3] Bao Y. W. Chau A. C. M. Chiu P. K. Shea Y. F. Kwan J. S. K. Chan F. H. W.. (2021). Heterogeneity of amyloid binding in cognitively impaired patients consecutively recruited from a memory clinic: evaluating the utility of quantitative 18F-Flutemetamol PET-CT in discrimination of mild cognitive impairment from Alzheimer's disease and other dementias. J. Alzheimers Dis. 79, 819–832. doi: 10.3233/JAD-200890, PMID: 33361593 PMC7902948

[ref4] Benseny-Cases N. Klementieva O. Cotte M. Ferrer I. Cladera J. (2014). Microspectroscopy (muFTIR) reveals co-localization of lipid oxidation and amyloid plaques in human Alzheimer disease brains. Anal. Chem. 86, 12047–12054. doi: 10.1021/ac502667b, PMID: 25415602

[ref5] Benyard B. Nanga R. P. R. Wilson N. E. Thakuri D. Jacobs P. S. Swain A.. (2023). In vivo reproducibility of 3D relayed NOE in the healthy human brain at 7 T. Magn. Reson. Med. 89, 2295–2304. doi: 10.1002/mrm.29600, PMID: 36744726 PMC10078808

[ref6] Bouhrara M. Reiter D. A. Bergeron C. M. Zukley L. M. Ferrucci L. Resnick S. M.. (2018). Evidence of demyelination in mild cognitive impairment and dementia using a direct and specific magnetic resonance imaging measure of myelin content. Alzheimers Dement. 14, 998–1004. doi: 10.1016/j.jalz.2018.03.007, PMID: 29679574 PMC6097903

[ref7] Breitling J. Deshmane A. Goerke S. Korzowski A. Herz K. Ladd M. E.. (2019). Adaptive denoising for chemical exchange saturation transfer MR imaging. NMR Biomed. 32:e4133. doi: 10.1002/nbm.4133, PMID: 31361064

[ref8] Chandra A. Dervenoulas G. Politis M. Alzheimer’s Disease Neuroimaging Initiative (2019). Magnetic resonance imaging in Alzheimer's disease and mild cognitive impairment. J. Neurol. 266, 1293–1302. doi: 10.1007/s00415-018-9016-3, PMID: 30120563 PMC6517561

[ref9] Chen G. F. Xu T. H. Yan Y. Zhou Y. R. Jiang Y. Melcher K.. (2017). Amyloid beta: structure, biology and structure-based therapeutic development. Acta Pharmacol. Sin. 38, 1205–1235. doi: 10.1038/aps.2017.28, PMID: 28713158 PMC5589967

[ref10] Cohen O. Huang S. McMahon M. T. Rosen M. S. Farrar C. T. (2018). Rapid and quantitative chemical exchange saturation transfer (CEST) imaging with magnetic resonance fingerprinting (MRF). Magn. Reson. Med. 80, 2449–2463. doi: 10.1002/mrm.27221, PMID: 29756286 PMC6234098

[ref11] Cunnane S. C. Chouinard-Watkins R. Castellano C. A. Barberger-Gateau P. (2013). Docosahexaenoic acid homeostasis, brain aging and Alzheimer's disease: can we reconcile the evidence? Prostaglandins Leukot. Essent. Fatty Acids 88, 61–70. doi: 10.1016/j.plefa.2012.04.006, PMID: 22575581

[ref12] Dean D. C. Hurley S. A. Kecskemeti S. R. O’Grady J. P. Canda C. Davenport-Sis N. J.. (2017). Association of Amyloid Pathology with Myelin Alteration in preclinical Alzheimer disease. JAMA Neurol. 74, 41–49. doi: 10.1001/jamaneurol.2016.3232, PMID: 27842175 PMC5195903

[ref13] Dorr A. E. Lerch J. P. Spring S. Kabani N. Henkelman R. M. (2008). High resolution three-dimensional brain atlas using an average magnetic resonance image of 40 adult C57Bl/6J mice. NeuroImage 42, 60–69. doi: 10.1016/j.neuroimage.2008.03.037, PMID: 18502665

[ref14] Dou W. Lin C. E. Ding H. Shen Y. Dou C. Qian L.. (2019). Chemical exchange saturation transfer magnetic resonance imaging and its main and potential applications in pre-clinical and clinical studies. Quant. Imaging Med. Surg. 9, 1747–1766. doi: 10.21037/qims.2019.10.03, PMID: 31728316 PMC6828581

[ref15] Downes N. Mullins P. (2014). The development of myelin in the brain of the juvenile rat. Toxicol. Pathol. 42, 913–922. doi: 10.1177/0192623313503518, PMID: 24129760

[ref16] Faizy T. D. Thaler C. Broocks G. Flottmann F. Leischner H. Kniep H.. (2020). The myelin water fraction serves as a marker for age-related myelin alterations in the cerebral white matter – a multiparametric MRI aging study. Front. Neurosci. 14:136. doi: 10.3389/fnins.2020.00136, PMID: 32153358 PMC7050496

[ref17] Gong Z. Bilgel M. Kiely M. Triebswetter C. Ferrucci L. Resnick S. M.. (2023). Lower myelin content is associated with more rapid cognitive decline among cognitively unimpaired individuals. Alzheimers Dement. 19, 3098–3107. doi: 10.1002/alz.12968, PMID: 36720000 PMC10387505

[ref18] Heo H. Y. Zhang Y. Lee D. H. Jiang S. Zhao X. Zhou J. (2017). Accelerating chemical exchange saturation transfer (CEST) MRI by combining compressed sensing and sensitivity encoding techniques. Magn. Reson. Med. 77, 779–786. doi: 10.1002/mrm.26141, PMID: 26888295 PMC4988943

[ref19] Huang J. Lai J. H. C. Tse K. H. Cheng G. W. Y. Liu Y. Chen Z.. (2022). Deep neural network based CEST and AREX processing: application in imaging a model of Alzheimer's disease at 3 T. Magn. Reson. Med. 87, 1529–1545. doi: 10.1002/mrm.29044, PMID: 34657318

[ref20] Igarashi K. M. (2023). Entorhinal cortex dysfunction in Alzheimer's disease. Trends Neurosci. 46, 124–136. doi: 10.1016/j.tins.2022.11.006, PMID: 36513524 PMC9877178

[ref21] Jones C. K. Huang A. Xu J. Edden R. A. Schar M. Hua J.. (2013). Nuclear Overhauser enhancement (NOE) imaging in the human brain at 7T. NeuroImage 77, 114–124. doi: 10.1016/j.neuroimage.2013.03.047, PMID: 23567889 PMC3848060

[ref22] Kang J. Rivest S. (2012). Lipid metabolism and neuroinflammation in Alzheimer's disease: a role for liver X receptors. Endocr. Rev. 33, 715–746. doi: 10.1210/er.2011-1049, PMID: 22766509

[ref23] Kao Y. A.-O. Ho P. C. Tu Y. K. Jou I. M. Tsai K. A.-O. (2020). Lipids and Alzheimer's disease. Int J Mol Sci 21:1505. doi: 10.3390/ijms21041505, PMID: 32098382 PMC7073164

[ref24] Kheterpal I. Wetzel R. Cook K. D. (2003). Enhanced correction methods for hydrogen exchange-mass spectrometric studies of amyloid fibrils. Protein Sci. 12, 635–643. doi: 10.1110/ps.0225703, PMID: 12592034 PMC2312450

[ref25] Kim M. Gillen J. Landman B. A. Zhou J. van Zijl P. C. (2009). Water saturation shift referencing (WASSR) for chemical exchange saturation transfer (CEST) experiments. Magn. Reson. Med. 61, 1441–1450. doi: 10.1002/mrm.21873, PMID: 19358232 PMC2860191

[ref26] Knopman D. S. Amieva H. Petersen R. C. Chetelat G. Holtzman D. M. Hyman B. T.. (2021). Alzheimer disease. Nat. Rev. Dis. Primers. 7:33. doi: 10.1038/s41572-021-00269-y, PMID: 33986301 PMC8574196

[ref27] Kofeler H. C. Fauland A. Rechberger G. N. Trotzmuller M. (2012). Mass spectrometry based lipidomics: an overview of technological platforms. Meta 2, 19–38. doi: 10.3390/metabo2010019, PMID: 24957366 PMC3901195

[ref28] Lim S.-H. Lee J. Jung S. Kim B. Rhee H. Y. Oh S.-H.. (2022). Myelin-weighted imaging presents reduced apparent myelin water in patients with Alzheimer&rsquo;s disease. Diagnostics 12:446. doi: 10.3390/diagnostics12020446, PMID: 35204537 PMC8871299

[ref29] Marcus C. Mena E. Subramaniam R. M. (2014). Brain PET in the diagnosis of Alzheimer's disease. Clin. Nucl. Med. 39, e413–e426. doi: 10.1097/RLU.0000000000000547, PMID: 25199063 PMC4332800

[ref30] Michel V. Yuan Z. Ramsubir S. Bakovic M. (2006). Choline transport for phospholipid synthesis. Exp. Biol. Med. (Maywood) 231, 490–504. doi: 10.1177/153537020623100503, PMID: 16636297

[ref31] Mielke M. M. Okonkwo O. C. Oishi K. Mori S. Tighe S. Miller M. I.. (2012). Fornix integrity and hippocampal volume predict memory decline and progression to Alzheimer's disease. Alzheimers Dement. 8, 105–113. doi: 10.1016/j.jalz.2011.05.2416, PMID: 22404852 PMC3305232

[ref32] Mrdenovic D. Su Z. Kutner W. Lipkowski J. Pieta P. (2020). Alzheimer's disease-related amyloid beta peptide causes structural disordering of lipids and changes the electric properties of a floating bilayer lipid membrane. Nanoscale Adv. 2, 3467–3480. doi: 10.1039/D0NA00292E, PMID: 36134289 PMC9417616

[ref33] Ni R. (2021). Magnetic resonance imaging in animal models of Alzheimer’s disease amyloidosis. Int. J. Mol. Sci. 22:12768. doi: 10.3390/ijms222312768, PMID: 34884573 PMC8657987

[ref34] Orzylowska A. Oakden W. (2021). Saturation transfer MRI for detection of metabolic and microstructural impairments underlying neurodegeneration in Alzheimer's disease. Brain Sci. 12:53. doi: 10.3390/brainsci12010053, PMID: 35053797 PMC8773856

[ref35] Prasad M. R. Lovell M. A. Yatin M. Dhillon H. Markesbery W. R. (1998). Regional membrane phospholipid alterations in Alzheimer's disease. Neurochem. Res. 23, 81–88. doi: 10.1023/A:1022457605436, PMID: 9482271

[ref36] Sanchez-Molina P. Kreuzer M. Benseny-Cases N. Valente T. Almolda B. Gonzalez B.. (2020). From mouse to human: comparative analysis between Grey and white matter by synchrotron-Fourier transformed infrared microspectroscopy. Biomol. Ther. 10:1099. doi: 10.3390/biom10081099, PMID: 32722088 PMC7464184

[ref37] Sasaguri H. Nilsson P. Hashimoto S. Nagata K. Saito T. De Strooper B.. (2017). APP mouse models for Alzheimer's disease preclinical studies. EMBO J. 36, 2473–2487. doi: 10.15252/embj.201797397, PMID: 28768718 PMC5579350

[ref38] Tanveer M. Richhariya B. Khan R. U. Rashid A. H. Khanna P. Prasad M.. (2020). Machine learning techniques for the diagnosis of Alzheimer’s disease. ACM Trans. Multimed. Comput. Commun. Appl. 16, 1–35. doi: 10.1145/3344998

[ref39] Van Zijl P. C. Yadav N. N. (2011). Chemical exchange saturation transfer (CEST): what is in a name and what isn't? Magn. Reson. Med. 65, 927–948. doi: 10.1002/mrm.22761, PMID: 21337419 PMC3148076

[ref40] Vercauteren T. Pennec X. Perchant A. Ayache N. (2009). Diffeomorphic demons: efficient non-parametric image registration. NeuroImage 45, S61–S72. doi: 10.1016/j.neuroimage.2008.10.040, PMID: 19041946

[ref41] Wells J. A. O'Callaghan J. M. Holmes H. E. Powell N. M. Johnson R. A. Siow B.. (2015). In vivo imaging of tau pathology using multi-parametric quantitative MRI. NeuroImage 111, 369–378. doi: 10.1016/j.neuroimage.2015.02.023, PMID: 25700953 PMC4626540

[ref42] Zaiss M. Schmitt B. Bachert P. (2011). Quantitative separation of CEST effect from magnetization transfer and spillover effects by Lorentzian-line-fit analysis of z-spectra. J. Magn. Reson. 211, 149–155. doi: 10.1016/j.jmr.2011.05.001, PMID: 21641247

[ref43] Zhang F. Pan B. Shao P. Liu P. Alzheimer's Disease Neuroimaging I Australian Imaging Biomarkers Lifestyle flagship study of ageing. (2022). A single model deep learning approach for Alzheimer's disease diagnosis. Neuroscience 491, 200–214. doi: 10.1016/j.neuroscience.2022.03.026, PMID: 35398507

[ref44] Zhao Y. Sun C. Zu Z. (2023). Assignment of molecular origins of NOE signal at-3.5 ppm in the brain. Magn. Reson. Med. 90, 673–685. doi: 10.1002/mrm.29643, PMID: 36929814 PMC10644915

[ref45] Zheng H. Zhou Q. Du Y. Li C. Xu P. Lin L.. (2018). The hypothalamus as the primary brain region of metabolic abnormalities in APP/PS1 transgenic mouse model of Alzheimer's disease. Biochim. Biophys. Acta Mol. basis Dis. 1864, 263–273. doi: 10.1016/j.bbadis.2017.10.028, PMID: 29107091

[ref46] Zhou J. Heo H. Y. Knutsson L. van Zijl P. C. M. Jiang S. (2019). APT-weighted MRI: techniques, current neuro applications, and challenging issues. J. Magn. Reson. Imaging 50, 347–364. doi: 10.1002/jmri.26645, PMID: 30663162 PMC6625919

